# A Comprehensive Study to Identify Major Metabolites of an Amoxicillin–Sulbactam Hybrid Molecule in Rats and Its Metabolic Pathway Using UPLC-Q-TOF-MS/MS

**DOI:** 10.3390/metabo12070662

**Published:** 2022-07-18

**Authors:** Fei-Ke Zhao, Ren-Bin Shi, Yu-Bin Sun, Shuang-Yun Yang, Liang-Zhu Chen, Bing-Hu Fang

**Affiliations:** 1 National Laboratory of Safety Evaluation (Environmental Assessment) of Veterinary Drugs, South China Agricultural University, Guangzhou 510642, China; zfk160099@163.com (F.-K.Z.); i76019@163.com (R.-B.S.); yang_syun@163.com (S.-Y.Y.); 2 Shenzhen Institute for Drug Control, Shenzhen 518057, China; 18826231020@163.com; 3 Guangdong Dahuanong Animal Health Products Co., Ltd., Yunfu 527400, China; che_lizh@163.com; 4 College of Veterinary Medicine, South China Agricultural University, Guangzhou 510630, China

**Keywords:** hybrid molecule, amoxicillin, sulbactam, drug metabolism, ultrahigh performance liquid chromatography–quadrupole time-of-flight tandem mass spectrometry

## Abstract

Amoxicillin and sulbactam are widely used compound drugs in animal food. The amoxicillin–sulbactam hybrid molecule can achieve better curative effects through the combination of the two drugs. However, its pharmacokinetic behavior needs to be explored. In this study, a randomized crossover experiment was performed to investigate the metabolism of the novel amoxicillin–sulbactam hybrid molecule in rats after gastric administration. Ultrahigh performance liquid chromatography–quadrupole time-of-flight tandem mass spectrometry (UPLC-Q-TOF-MS/MS) was used to isolate and to identify the metabolites in rats. Amoxicillin, amoxicilloic acid, amoxicillin diketopiperazine, and sulbactam were eventually detected in the plasma, liver, urine, and kidneys; no hybrid molecules and their metabolites were detected in feces. The in vivo metabolism results showed that the hybrid molecule was absorbed into the body in the intestine, producing amoxicillin and sulbactam, then amoxicillin was partially metabolized to amoxicilloic acid and amoxicillin diketopiperazine, which are eventually excreted in the urine by the kidneys. In this study, four major metabolites of the amoxicillin–sulbactam hybrid molecule were identified and their metabolic pathways were speculated, which provided scientific data for understanding the metabolism of the hybrid molecule and for its clinical rational use.

## 1. Introduction

Antibiotics are an effective means to prevent diseases and to improve feed efficiency for agricultural animals [1]. Amoxicillin is a semi-synthetic penicillin derivative that is widely used in veterinary and in human medicine owing to its broad-spectrum antimicrobial activity against Gram-negative and -positive pathogens [2,3]. However, amoxicillin monotherapy has proven less effective in recent years owing to the widespread emergence of multidrug-resistant bacteria [4,5,6]. Accordingly, amoxicillin/sulbactam combinations have become common compound preparations in clinical studies. It is important to note that the combination of sulbactam with β-lactam antibiotics does not improve its pharmacokinetic characteristics in terms of its poor oral absorption, so it is necessary to develop new and improved drugs through new techniques.

Drug splicing refers to linking different drugs or active ingredients of drugs together through chemical bonds to form new drugs with multiple targets or mechanisms of action. There are two common drug-splicing strategies: One is where the chemical bonds formed by the splicing are hydrolyzed by specific enzymes in vivo to release the original active ingredients, thereby playing a dual role [7,8]. The other is that the chemical bonds formed by splicing are not hydrolyzed by enzymes in vivo and function as new hybrid molecules [9].

Sultamicillin is a diester compound formed by combining ampicillin and sulbactam via a methylene group, it has been clinically proven to be an orally effective combination drug [10]. Sultamicillin is hydrolyzed by enteric esterase after oral administration, releasing equal molar amounts of ampicillin and sulbactam for absorption by the body [11]. This expands the antibacterial spectrum of ampicillin, especially strengthening its antibacterial activity against β-lactamase-producing bacteria [12].

**Figure 1:**
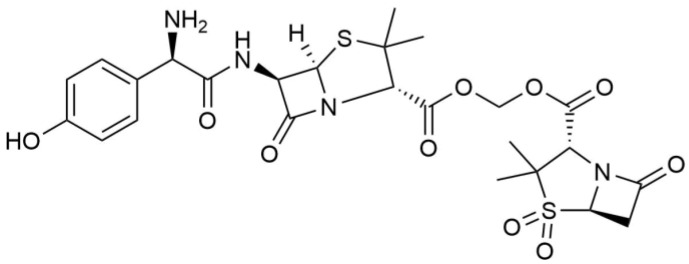
Chemical structure of AS.

In the synthesis method based on sultazicillin [13,14], we linked amoxicillin and sulbactam through a methylene bridge and synthesized a novel amoxicillin–sulbactam hybrid molecule (AS, Figure 1). This recovers the antibacterial activity of amoxicillin against resistant bacteria and optimizes the pharmacokinetic properties of sulbactam.

Drug metabolism refers to changes in the physical properties and chemical structure of a drug under the action of biological factors in the body [15,16]. Accordingly, drug metabolism is extremely important to the pharmacology and toxicology of a substance [17]. Drug metabolism is usually divided into two phases: Phase I involves bioconversion under the action of enzymes and typically includes processes such as oxidation, reduction, hydrolysis, and hydroxylation, while Phase II involves covalent binding to form complexes with a high polarity that are thus easily secreted [18,19]. The metabolic transformation of drugs plays an important role in their overall medicinal properties as well as their pharmacological and toxicological characteristics [20,21]. Accordingly, the main reason for identifying drug metabolites in clinical research is to ensure their safety [22].

Gas chromatography, liquid chromatography, and tandem mass spectrometry are important methods used to identify different metabolites in drug analysis [23,24,25,26]. With the advent of liquid chromatography–mass spectrometry (LC–MS), metabolites can be fully separated, and even trace substances can be detected. Accordingly, LC–MS-based methods have become the most effective and widely used analytical tools for the identification of drug metabolites [27,28].

For instance, using human liver microsomes incubated with amitriptyline and verapamil as test samples, Rousu et al. found and preliminarily identified 97 metabolites while comparing the metabolite screening characteristics of triple quadrupole, mixed linear ion trap triple quadrupole, time-of-flight, and combined mass spectrometries. The authors reported that time-of-flight mass spectrometry (TOF–MS) was the only method that detected all the metabolites, and it was also the quickest [29]. The applicability of different types of mass spectrometry to metabolite mapping varies greatly [30]. The advantage of TOF–MS is that it can obtain detailed information on biotransformation sites and has high sensitivity, quality, accuracy, chromatographic compatibility, and data collection rates [31]. In addition, TOF–MS can collect more qualitative and quantitative information about drugs and their metabolites, and even endogenous biomarkers, from the same sample at the same time, simplifying the screening process. Accordingly, it is a very powerful tool for drug discovery and development [32,33].

In this study, the metabolism of AS in rats was studied by ultrahigh performance liquid chromatography–quadrupole time-of-flight tandem mass spectrometry (UPLC-Q-TOF-MS/MS), and the metabolites of the hybrid molecule were identified, providing scientific data for rational clinical use of the compound.

## 2. Materials and Methods

### 2.1. Materials

#### 2.1.1. Drugs and Reagents

Amoxicillin (87.00%, Batch No. 130409-201913) was obtained from the China National Institute for Food and Drug Control (Beijing, China).

Sulbactam (98.54%, Lot No. DM21022603) was obtained from Guangzhou Juanmu Biotechnology Co., Ltd. (Guangzhou, China).

Amoxicillin diketone piperazine (93.68%, Lot No. DM20051896) was obtained from Guangzhou Juanmu Biotechnology Co., Ltd. (Guangzhou, China). Amoxicilloic acid (99.18%, Lot A634265) was obtained from Toronto Research Chemicals (Wuhan, China).

AS (content detected by HPLC: 93.00%) (amoxicillin content: 55.50%; sulbactam content: 35.37%). The NMR spectrum of AS is: 1H NMR (600 MHz, DMSO-d6) δ 9.82 (s, 1H, OH), 9.28 (d, J = 7.5 Hz, 1H, NH), 8.57 (s, 1H, NH), 7.28 (d, J = 8.6 Hz, 2H), 6.80 (d, J = 8.6 Hz, 2H), 5.91 (q, J = 6.1 Hz, 2H, OCH2O), 5.59 (t, J = 4.8 Hz, 1H), 5.45 (d, J = 4.1 Hz, 1H), 5.20 (dd, J = 4.6, 1.8 Hz, 1H), 4.95 (s, 1H), 4.56 (s, 1H), 4.42 (s, 1H), 4.03 (q, J = 7.1 Hz, 1H), 3.73–3.65 (m, 1H), 1.49 (s, 3H), 1.46 (s, 3H), 1.36 (s, 3H), 1.35 (s, 3H).

Acetonitrile, methanol, and formic acid were obtained at chromatographic grade from Thermo Fisher Scientific (China) Co., Ltd. (Shanghai, China).

#### 2.1.2. Instruments

The following equipment was used in this study:

A UPLC1290-6540B Q-TOF; a 1290 ULTRA high-pressure liquid chromatography system; a 6540B Q-TOF quadrupole tandem TOF-MASS spectrometry system with AJS, ESI, and APCI sources; a Mass Hunter workstation, and Metabolites ID software (Agilent Technologies, Ltd.) (Beijing, China).

A UPLC 1290-6470A ultrahigh performance liquid chromatography-triple quadrupole mass spectrometer equipped with an ultrahigh-pressure binary gradient pump, an ultra-efficient automatic sampler, an ultra-efficient column temperature chamber, and triple quadrupole mass spectrometer (Agilent Technologies Co., Ltd., Beijing, China).

A Waters UPLC (I-class) ultrahigh performance liquid chromatographer with a PDA detector (Waters Corporation, Milford, MA, USA) (Beijing, China).

A Waters Xevo TQ-S Micro with a Tipped XBOBbie Dynamic Range (XDR) detector and an electrospray ion source (ESI) source.

An Oasis HLB solid-phase extraction column (200 mg/3 mL; Waters Corporation, Milford, MA, USA).

#### 2.1.3. Solution Preparation

Standard solutions: Accurately weigh 11.49 mg, 10.08 mg, and 10.67 mg of amoxicillin, amoxicilloic acid, and amoxicillin diketopiperazine standard in three 10 mL brown volumetric flasks, then add in acetonitrile/water (1:1, *v*/*v*), dissolve and dilute to volume, prepare 1.00 mg/mL standard stock solution of amoxicillin, amoxicilloic acid, and amoxicillin-diketopiperazine, and store at −75 °C for later use.

Sulbactam standard solution: The standard sulbactam (10.15 mg) was precisely weighed into a 10-mL brown volumetric flask and dissolved in ultrapure water to a volume of 10 mL to obtain a standard solution with a concentration of 1.00 mg/mL.

AS solution: The AS (107.53 mg) was weighed into a 10-mL brown volumetric flask and dissolved in ultrapure water to 10 mL to obtain a heterozygous molecular solution with a concentration of 10 mg/mL.

### 2.2. Metabolites of AS in Rats

#### 2.2.1. Animal Tests

Healthy male and female SD rats (SPF grade, SCXK (Liao) 2020-0001) weighing 180–220 g were used in this study. All animals were reared in standardized conditions with a relative humidity of 60%, temperature of 21 °C, and 12-h light/dark cycle and allowed free access to a standard diet and water. Animal experiments were conducted in strict agreement with protocols approved by the Institutional Animal Care and Use Committee of South China Agricultural University.

For sample collection, 28 rats were adaptively fed for 7 days, then fasted but allowed access to water for 12 h before the experiment. They were then randomly divided into seven groups (4 rats each: 2 males and 2 females), one control group (group A) and the other sample groups (group B), which were treated with 100 mg/kg b.w. (group A was given the same dose of normal saline). One of the groups was randomly selected to collect blood, urine, and feces at 0.5 h, 1 h, 2 h, 3 h, 6 h, and 12 h after administration. After that, the rat’s liver and kidneys of the group were harvested after death from excessive ether anesthesia. Blood samples were quickly added to heparin sodium and centrifuged at 1720 rcf and 4 °C for 10 min to separate the plasma. All plasma and urine samples in group A were absorbed in the same volume and combined with homogenization. All liver and kidney samples were pooled and homogenized, and the above steps were repeated for group B. The samples were stored at −20 °C.

#### 2.2.2. Sample Pretreatment

Plasma and urine: A 200-μL sample was accurately transferred to a 1.5-mL centrifuge tube, and 800 μL acetonitrile was added before vortexing for 2 min and then ultrasonication for 5 min. After being centrifuged at 10,700 rcf and 4 °C for 10 min, the supernatant was taken into a 2-mL centrifuge tube and dried with nitrogen at 40 °C. The residue was redissolved in 200 μL acetonitrile in water (30%) with vortexing for 2 min and then ultrasonication for 5 min, followed by centrifugation for 10 min at 10,700 rcf. The supernatant was filtered through a 0.22-μm organic membrane for UPLC-Q-TOF-MS/MS analysis.

Feces, liver, and kidneys: First, 1 g samples were accurately weighed into a 10 mL centrifuge tube, and 3 mL acetonitrile was added before vortexing for 1 min, ultrasonication for 5 min, and full oscillation for 5 min. The samples were then centrifuged for 10 min at 10,700 rcf and 4 °C. The supernatant was taken into a 5 mL centrifuge tube and blow-dried with nitrogen at 40 °C. Then, 1 mL ultrapure water was added to dissolve the residue, and 3 mL dichloromethane was added before the mixture was vortexed for 1 min and centrifuged again.

A Waters HLB solid-phase extraction column was activated with 3 mL methanol and ultrapure water successively, and the supernatant obtained by centrifugation was drawn onto the extraction column, which was rinsed with 1 mL ultrapure water and eluted with 2 mL 95% acetonitrile/water. The eluent was dried with nitrogen at 40 °C. Then, the residue was redissolved in 0.2 mL 30% acetonitrile, centrifuged at 10,700 rcf for 10 min, and the supernatant was filtered through a 0.22-μm organic filter membrane prior to UPLC-Q-TOF-MS/MS analysis.

#### 2.2.3. Liquid Chromatography Conditions

Separation was performed using an Agilent ECLIPSE PLUS C18 (2.1 × 100 mm, 1.8 μm) chromatographic column. The mobile phases were (A) acetonitrile and (B) 0.2% formic acid water. The flow rate was 300 μL/min, the column temperature was 40.00 °C, and the injection volume was 5 μL. Table 1 shows the gradient elution protocol.

#### 2.2.4. Mass Spectrometry Conditions

An ESI source was used in positive/negative ion mode; scan range: 100–1000 Da; atomization temperature: 300 °C; sheath gas: nitrogen; atomizing flow: 8 L/min; nebulizer: 45 PSIG; sheath gas temperature: 350 °C; sheath gas flow: 10 L/min; capillary voltage: 4000 V; nozzle voltage: 1000 V; fragmentor voltage: 130 V.

## 3. Results and Discussion

### 3.1. Metabolism of Heterozygous Molecules

**Figure 2:**
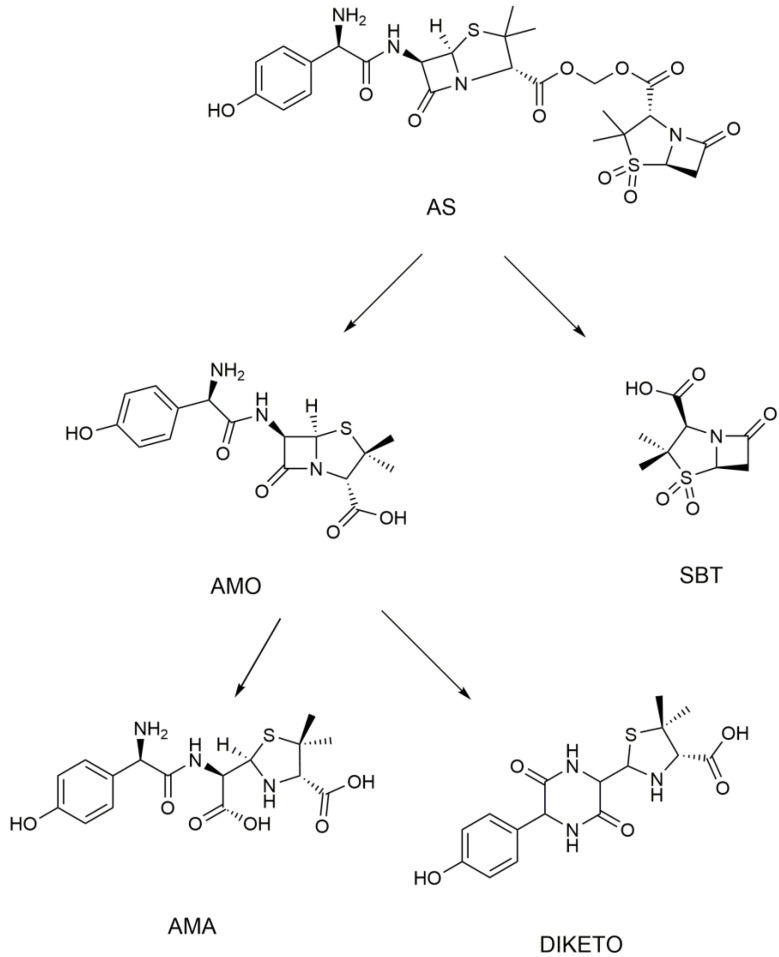
Metabolite-prediction diagram for AS.

The metabolites were preliminarily predicted based on the principle of splicing. There is only one hydroxyl difference between the spliced drug AS and Sultamicillin, so information on Sultamicillin metabolism in vivo could help us to preliminarily predict the metabolites. Sultamicillin is hydrolyzed into sulbactam and ampicillin by enterolactase in the intestinal wall after oral administration [11,34,35], so it is speculated that AS hydrolyzed in vivo to amoxicillin and sulbactam. Because amoxicillin is easily metabolized to amoxicilloic acid (AMA) and amoxicillin diketopiperazine (DIKETO) [36,37], it is speculated that some of the amoxicillin will be metabolized to these compounds in vivo. It has been reported that sulbactam exists mainly in the archetypal form in rats [38,39], so it is speculated that sulbactam exists in rats. The metabolite-prediction diagram for AS in rats is shown in Figure 2.

### 3.2. UPLC-Q-TOF-MS/MS Analysis of Standards

**Figure 3:**
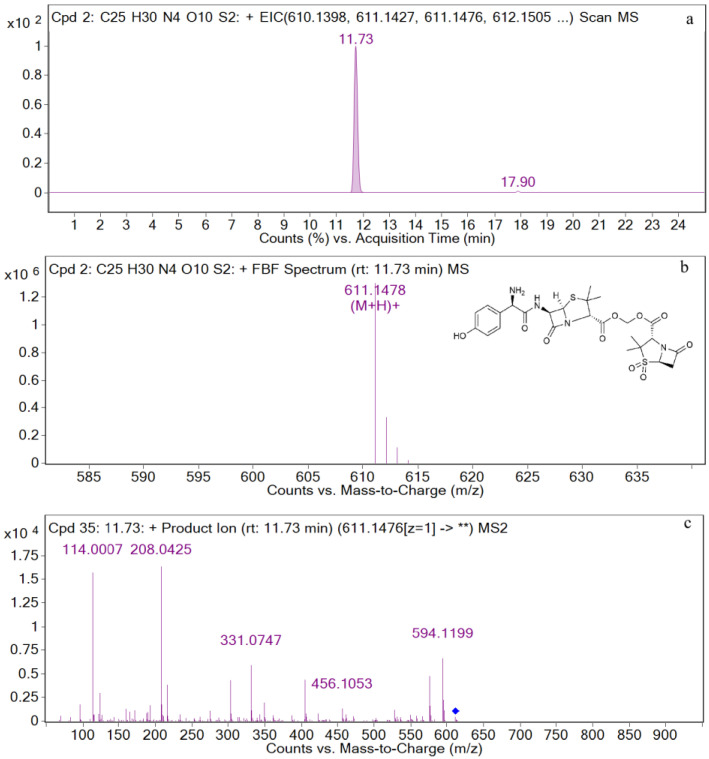
EIC and mass spectrometry results for AS. (**a**) EIC spectrometry results for AS; (**b**) Mass spectrum of AS; (**c**) Two-stage mass spectral data of AS.

The EIC and mass spectrometry results for AS are shown in Figure 3. The retention time is 11.73 min, and the ion peak is *m*/*z* 611.1478 [M+H]^+^. The fragment ions are *m*/*z* 594, *m*/*z* 456, *m*/*z* 331, *m*/*z* 208, and *m*/*z* 114. The characteristic ions are *m*/*z* 594, *m*/*z* 456, and *m*/*z* 331.

**Figure 4:**
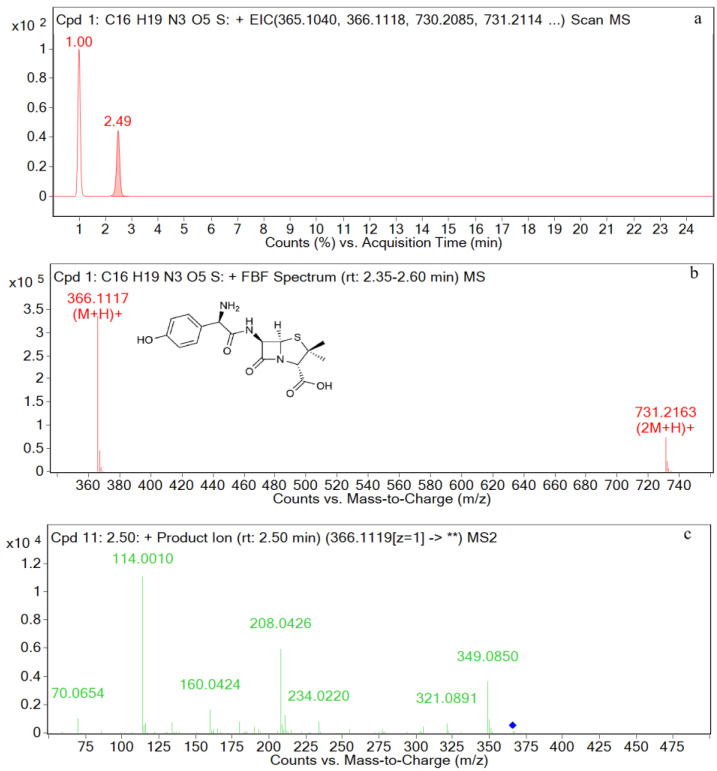
EIC and mass spectrometry results for amoxicillin. (**a**) EIC spectrometry results for amoxicillin; (**b**) Mass spectrum of amoxicillin; (**c**) Two-stage mass spectral data of amoxicillin.

The EIC and mass spectrometry results for amoxicillin are shown in Figure 4. The retention time is 2.5 min, and the ion peak is *m*/*z* 366.1117 [M+H]^+^. The fragment ions are *m*/*z* 349, *m*/*z* 321, *m*/*z* 234, *m*/*z* 208, *m*/*z* 160, *m*/*z* 114, and *m*/*z* 70. The characteristic ions are *m*/*z* 349, *m*/*z* 208, *m*/*z* 160, and *m*/*z* 114.

**Figure 5:**
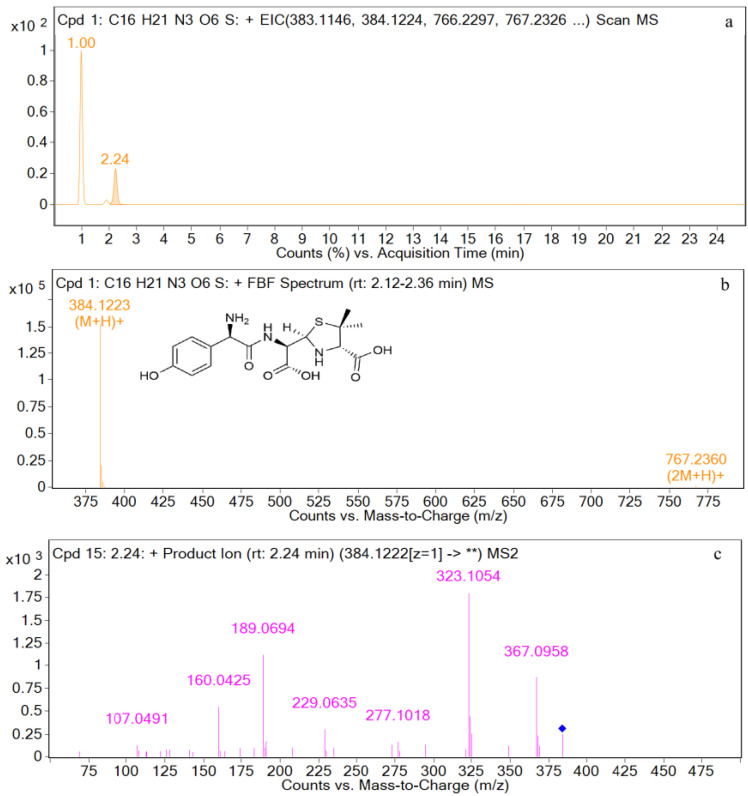
EIC and mass spectrometry results for amoxicilloic acid. (**a**) EIC spectrometry results for amoxicilloic acid; (**b**) Mass spectrum of amoxicilloic acid; (**c**) Two-stage mass spectral data of amoxicilloic acid.

The EIC and mass spectrometry results for amoxicilloic acid are shown in Figure 5. The retention time is 2.2 min, and the ion peak is *m*/*z* 384.1223 [M+H]^+^. The fragment ions are *m*/*z* 367, *m*/*z* 323, *m*/*z* 189, *m*/*z* 160, *m*/*z* 229, *m*/*z* 277, and *m*/*z* 107. The characteristic ions are *m*/*z* 367, *m*/*z* 323, and *m*/*z* 189.

**Figure 6:**
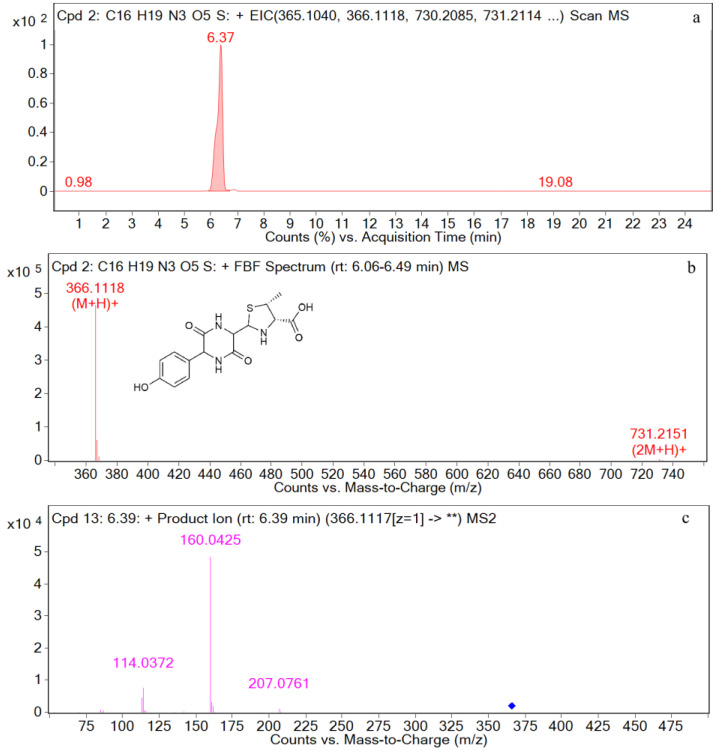
EIC and mass spectrometry results for amoxicillin diketopiperazine. (**a**): EIC spectrometry results for amoxicillin diketopiperazine; (**b**): Mass spectrum of amoxicillin diketopiperazine; (**c**): Two-stage mass spectral data of amoxicillin diketopiperazine.

The EIC and mass spectrometry results for amoxicillin diketopiperazine are shown in Figure 6. The retention time is 6.4 min, and the ion peak is *m*/*z* 366.1118 [M+H]^+^. The fragment ions are *m*/*z* 207, *m*/*z* 160, and *m*/*z* 114. The characteristic ions are *m*/*z* 207 and *m*/*z* 160.

**Figure 7:**
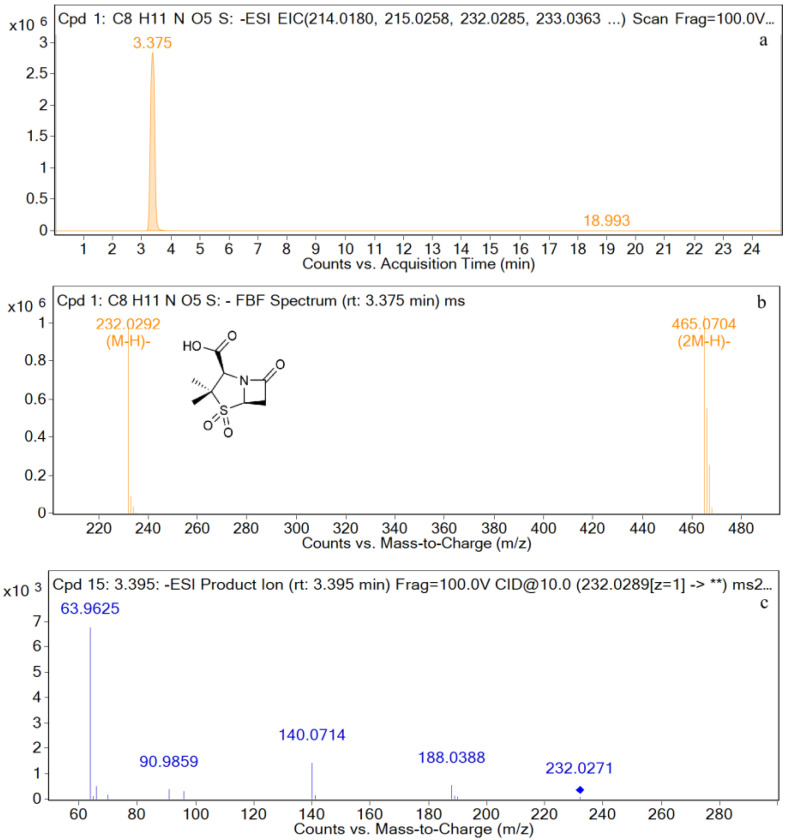
EIC and mass spectrometry results for Sulbactam. (**a**): EIC spectrometry results for sulbactam; (**b**): Mass spectrum of sulbactam; (**c**): Two-stage mass spectral data of sulbactam.

The EIC and mass spectrometry results for sulbactam are shown in Figure 7. The retention time is 3.4 min, and the ion peak is *m*/*z* 232.0292 [M−H]^−^. The fragment ions are *m*/*z* 188, *m*/*z* 140, *m*/*z* 91, and *m*/*z* 64. The characteristic ions are *m*/*z* 140 and *m*/*z* 64.

### 3.3. Metabolites in Different Tissues

**Figure 8:**
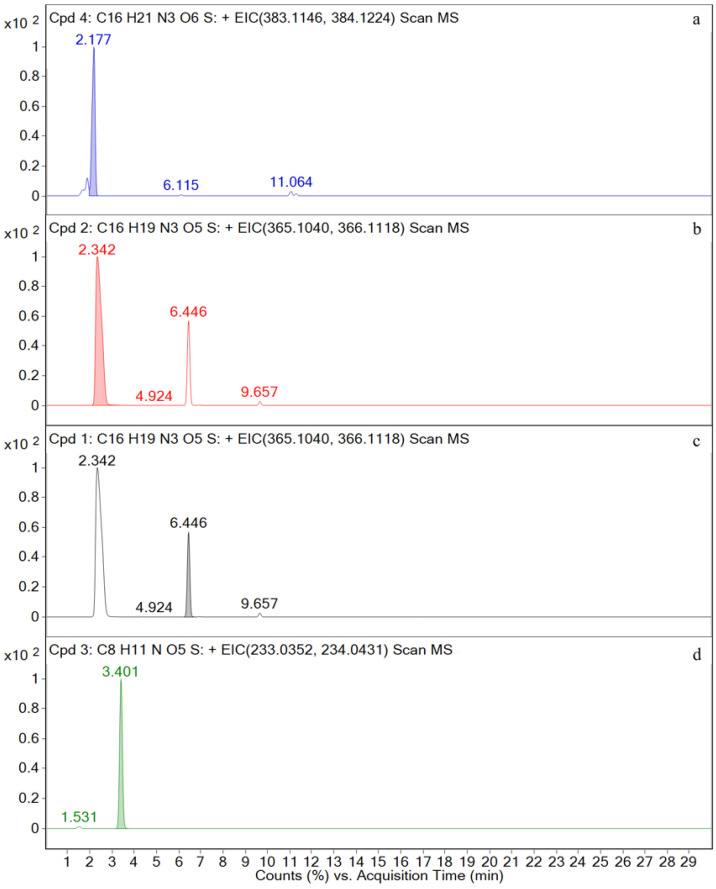
Ion chromatograms of urine samples. (**a**) M2; (**b**) M1; (**c**) M3; (**d**) M4.

**Figure A1:**
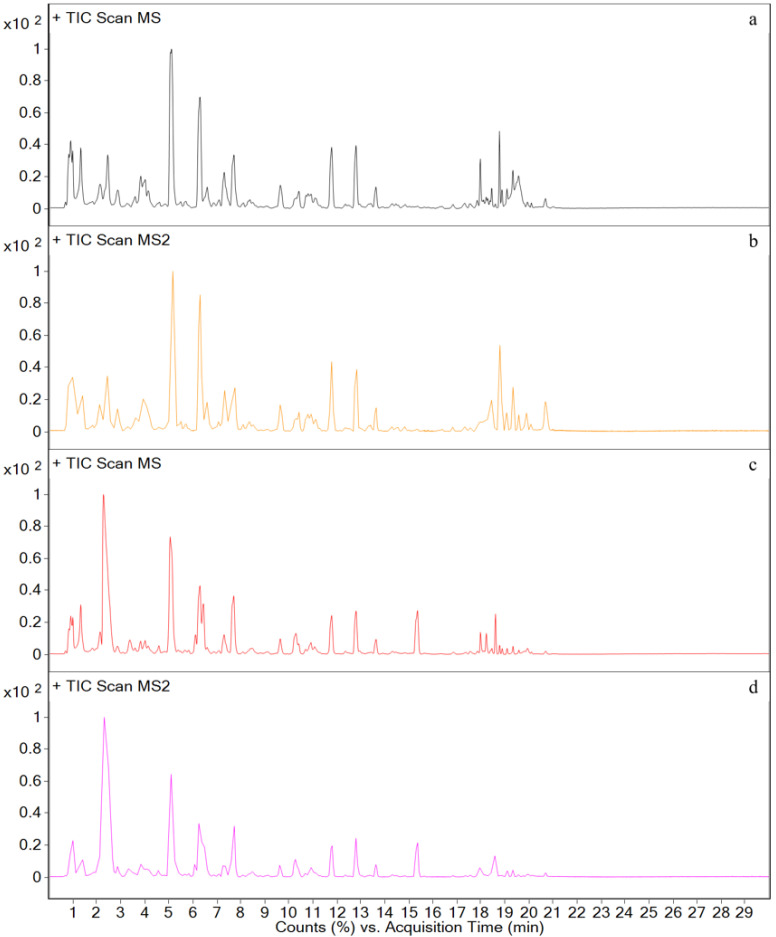
Total ion chromatograms of urine. (**a**,**b**) Blank urine; (**c**,**d**) Sample urine.

A total of four metabolites with retention times of 2.5, 2.2, 6.4, and 3.4 min, respectively, are detected in the samples. Their molecular weights are 365, 383, 365, and 233 Da, denoted as M1, M2, M3, and M4, respectively. No drug or metabolites are detected in the feces. The results for the urine, kidney, plasma, and liver are as follows:

M1, M2, M3, and M4 are detected in the urine samples. The EIC and total ion chromatogram (TIC) for the urine sample are shown in Figure 8 and Figure A1 in the Appendix A, respectively.

**Figure 9:**
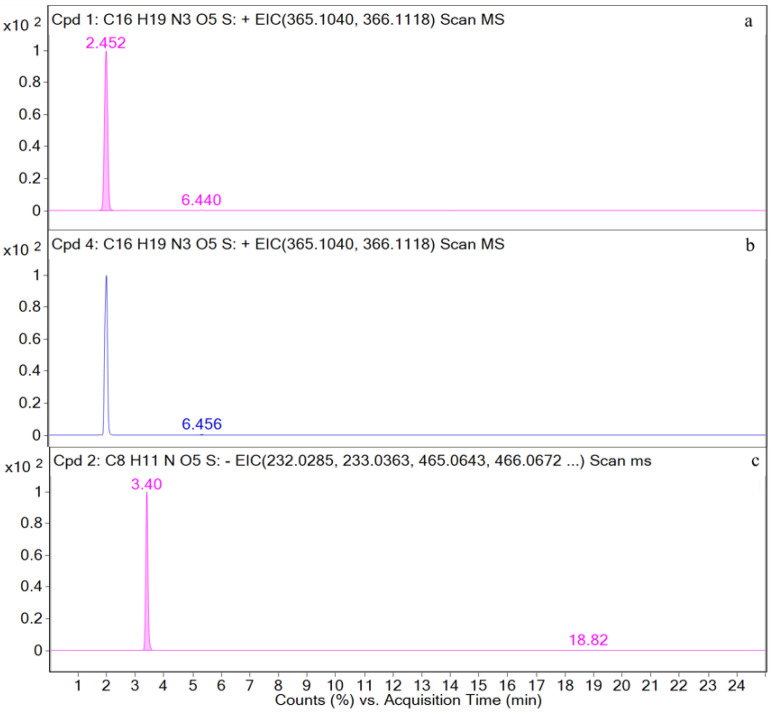
Ion chromatograms of kidney samples. (**a**) M1; (**b**) M3; (**c**) M4.

**Figure A2:**
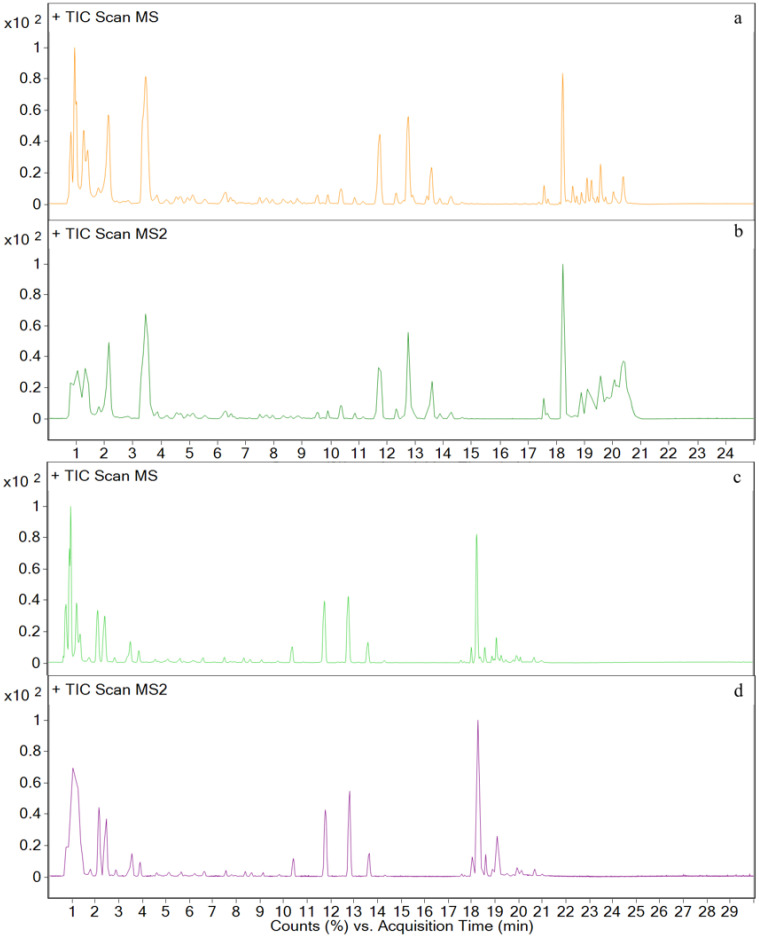
Total ion chromatograms of kidney. (**a**,**b**) Blank kidney; (**c**,**d**) Sample kidney.

M1, M3, and M4 are detected in the kidney samples. The EIC and TIC for the kidney sample are shown in Figure 9 and Figure A2 in the Appendix A, respectively.

**Figure 10:**
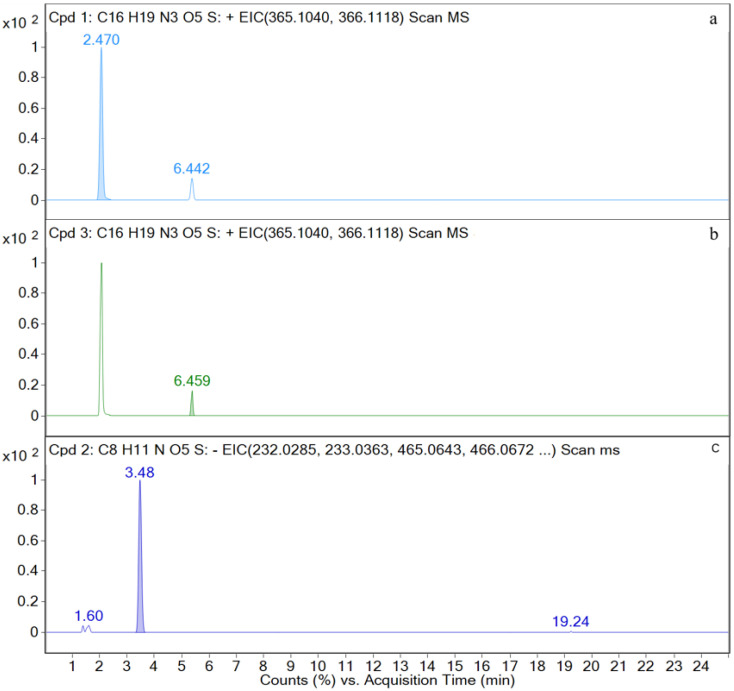
Ion chromatograms of plasma samples. (**a**) M1; (**b**) M3; (**c**) M4.

**Figure A3:**
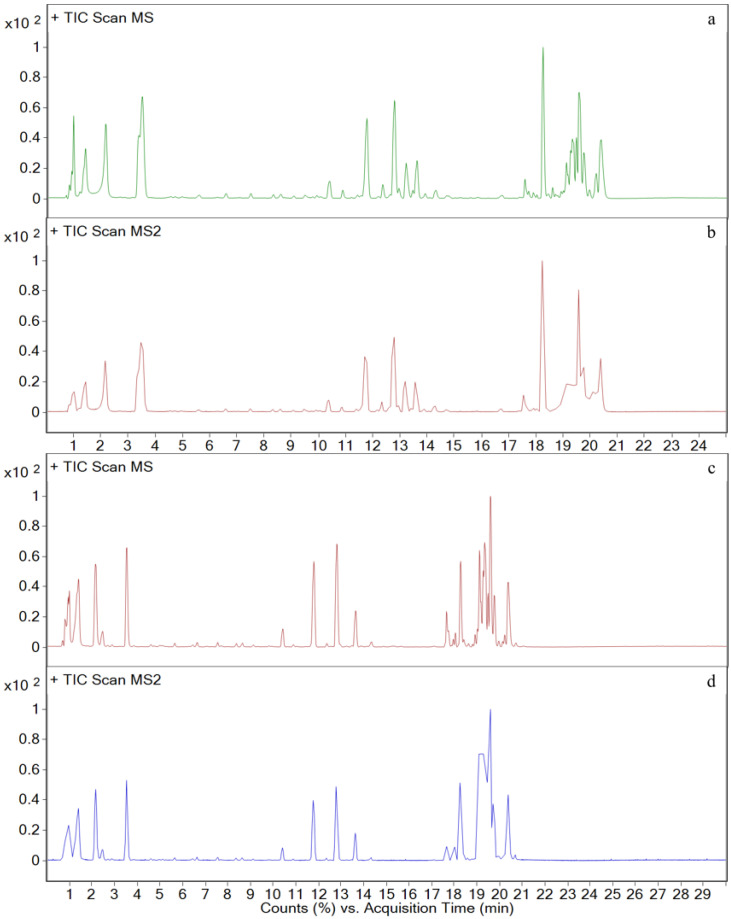
Total ion chromatograms of plasma. (**a**,**b**) Blank plasma; (**c**,**d**) Sample plasma.

M1, M3, and M4 are detected in the plasma samples. The EIC and TIC for the plasma sample are shown in Figure 10 and Figure A3 in the Appendix A, respectively.

**Figure 11:**
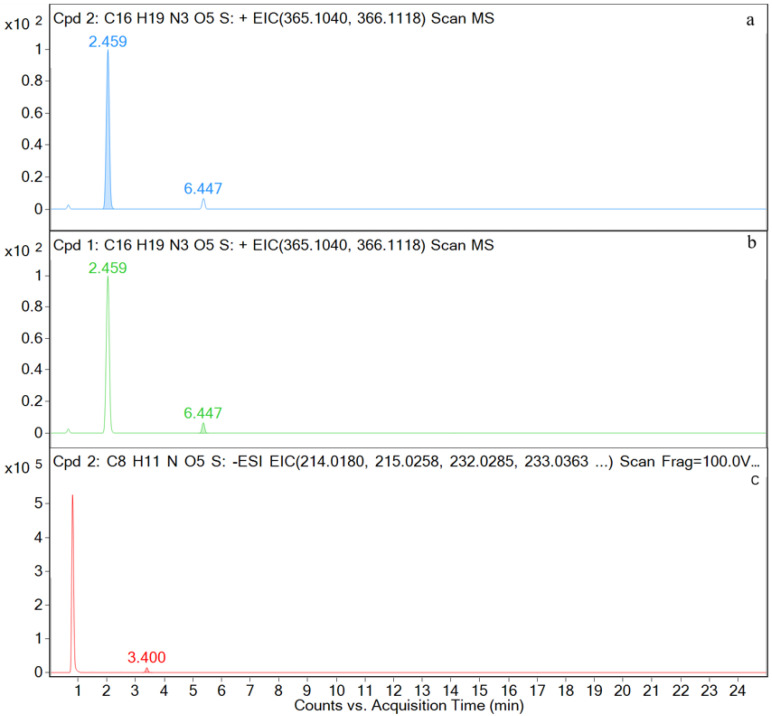
Ion chromatograms of liver samples. (**a**) M1; (**b**) M3; (**c**) M4.

**Figure A4:**
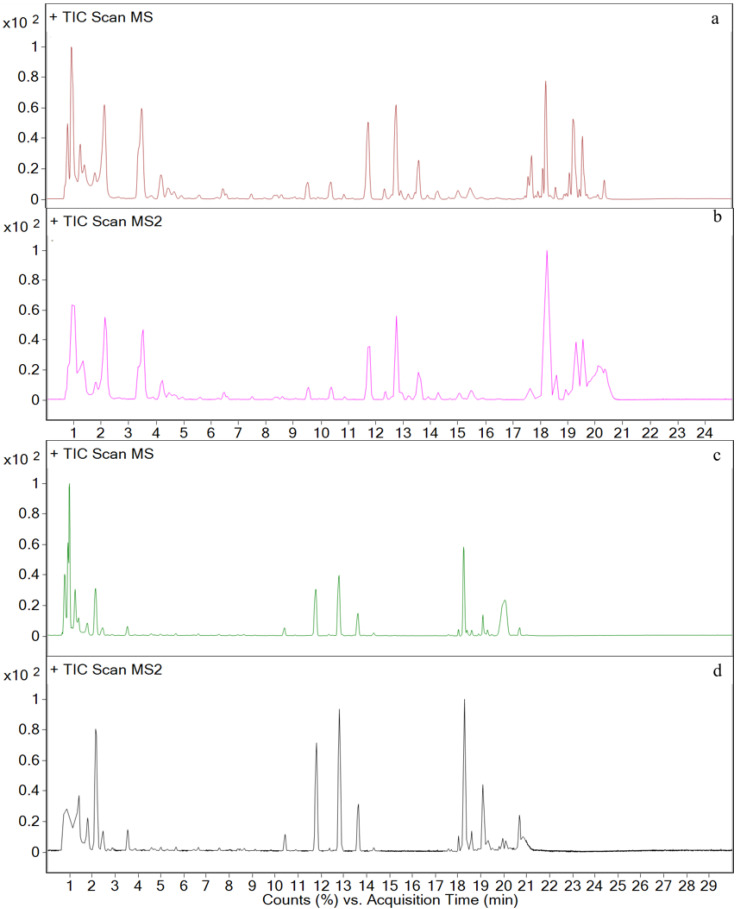
Total ion chromatograms of liver. (**a**,**b**) Blank liver; (**c**,**d**) Sample liver.

M1, M3, and M4 are detected in the liver sample. The EIC and TIC for the liver sample are shown in Figure 11 and Figure A4 in the Appendix A, respectively.

**Figure A5:**
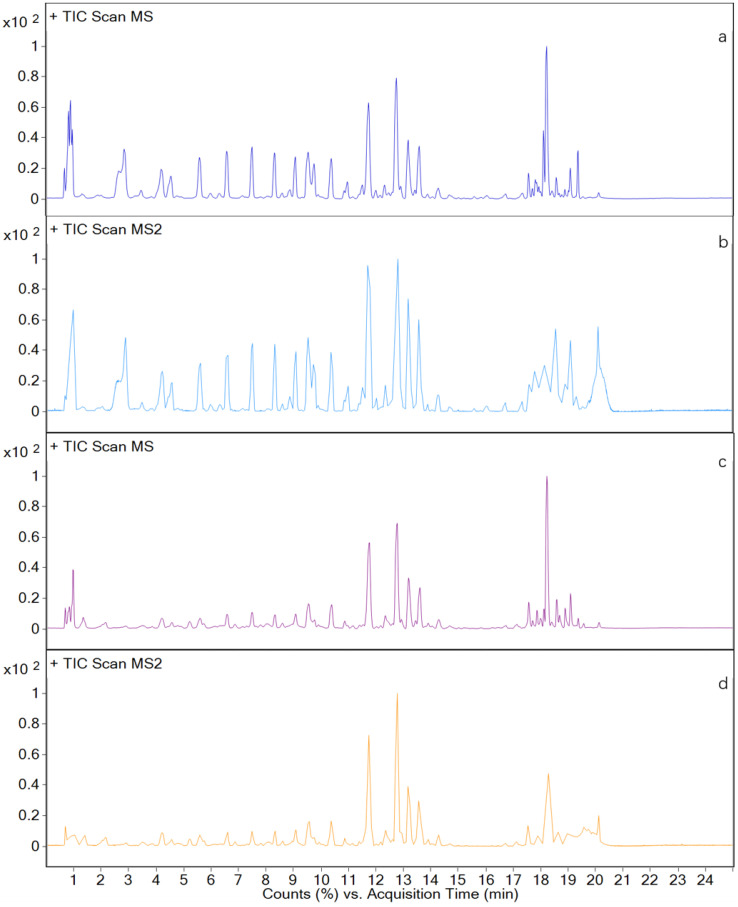
Total ion chromatograms of feces. (**a**,**b**) Blank feces; (**c**,**d**) Sample feces.

No drug or metabolites are detected in the feces. The total ion chromatogram (TIC) for the feces sample is shown in Figure A5 in the Appendix A.

**Figure 12:**
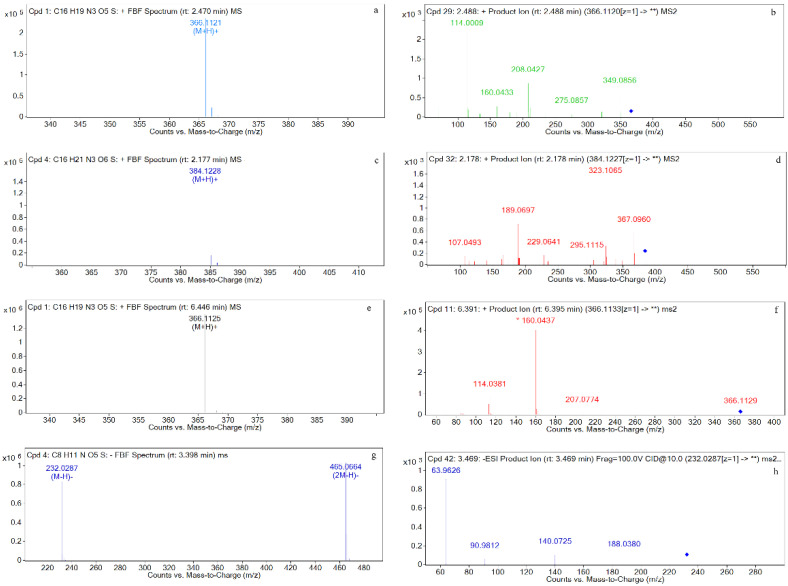
Mass spectra of four metabolites M1, M2, M3, and M4. (**a**) Mass spectrum of M1; (**b**) Two-stage mass spectral data of M1; (**c**) Mass spectrum of M2; (**d**) Two-stage mass spectral data of M2; (**e**) Mass spectrum of M3; (**f**) Two-stage mass spectral data of M3; (**g**) Mass spectrum of M4; (**h**) Two-stage mass spectral data of M4.

The mass spectra of four metabolites M1, M2, M3, and M4 are shown in Figure 12.

### 3.4. In Vivo Metabolism Results

The chromatographic (Figure 8, Figure 9, Figure 10 and Figure 11) and mass spectrometry data (Figure 12) for M1, M2, M3, and M4 are consistent with those of the amoxicillin, amoxicilloic acid, amoxicillin diketopiperazine, and sulbactam standards (Figure 4, Figure 5, Figure 6 and Figure 7), respectively. Thus, it can be determined that M1, M2, M3, and M4 are amoxicillin, amoxicilloic acid, amoxicillin diketopiperazine, and sulbactam, respectively. Table 2 shows the molecular formulae, retention times, measured molecular weights, mass errors, and fragment ion information for AS and its metabolites.

## 4. Discussion

Four main metabolites, M1, M2, M3, and M4, were identified in the tissues and the plasma. From their chromatograms and mass spectrograms (Figure 3, Figure 4, Figure 5, Figure 6 and Figure 7), the four main metabolites were identified as amoxicillin, amoxicilloic acid, amoxicillin diketopiperazine, and sulbactam. Below are details on the identification of each.

**Figure 13:**
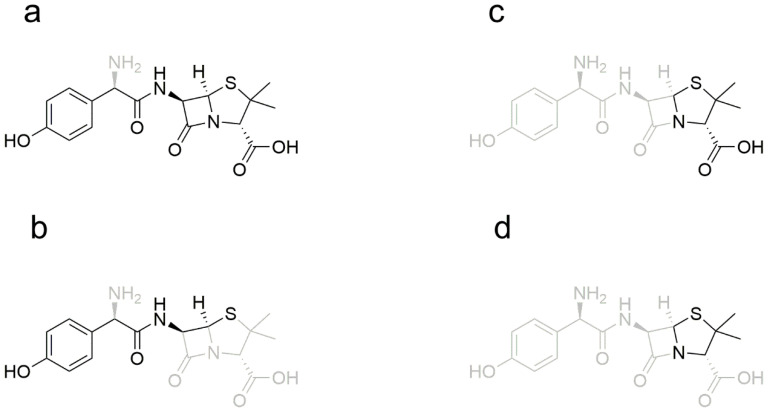
Possible structures of fragment ions in amoxicillin. (**a**) Possible structure of the *m*/*z* 349 fragment ion; (**b**) Possible structure of the *m*/*z* 208 fragment ion; (**c**) Possible structure of the *m*/*z* 160 fragment ion; (**d**) Possible structure of the *m*/*z* 114 fragment ion.

For M1 (retention time = 2.4 min), the parent ion in the primary mass spectrometry results is *m*/*z* 366 [M+H]^+^, and the molecular formula was predicted to be C_16_H_19_N_3_O_5_S by MSC software. From secondary mass spectrometry analysis, the main fragment ions are *m*/*z* 349, *m*/*z* 208, *m*/*z* 160, and *m*/*z* 114. Studies have found that there are three ring-opening modes for amoxicillin in mass spectrometry pyrolysis, all of which can generate fragment ions of *m*/*z* 114, among which fragment ions of *m*/*z* 349 can be used as characteristic fragments for qualitative and quantitative analysis of amoxicillin [40,41]. The retention time and characteristic ion fragments of M3 are also consistent with those of the amoxicillin standard, so it is speculated that M3 is amoxicillin. Suwanrumpha et al. explored the cleavage mode of amoxicillin and found that the loss of amino groups from the benzyl side chain is important for the initial fragment of cleavage, so fragment ions at *m*/*z* 349 are easily cleaved [41]. In the papers published so far, the formation of the ion at *m*/*z* 208 for amoxicillin was rationalized as a result of breaking two bonds of the β-lactam ring [42,43,44]. Jung et al., Freitas et al., and Nägele et al. believed that the fragment ion *m*/*z* 114 in amoxicillin was the loss of the carboxyl group of the fragment ion from *m*/*z* 160 [42,44,45]. The possible structures of the fragment ions are shown in Figure 13.

**Figure 14:**
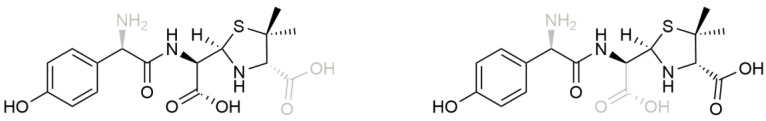
Two possible positions of fragment *m*/*z* 323 ions in amoxicilloic acid.

**Figure 15:**
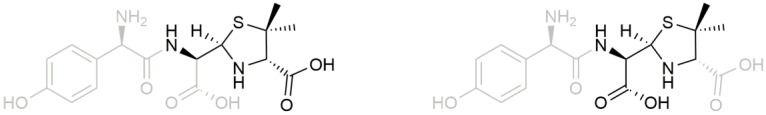
Two possible positions of fragment *m*/*z* 189 ions in amoxicilloic acid.

For M2 (retention time = 2.2 min), the parent ion *m*/*z* 384 [M+H]^+^ was identified in the first-level mass spectrometry, and the molecular formula was predicted to be C_16_H_21_N_3_O_6_S by MSC software. The secondary mass spectrometry analysis showed that the main fragment ions are *m*/*z* 323, and *m*/*z* 189. Amoxicilloic acid has been reported to have *m*/*z* 323 as the quantifying ion and *m*/*z* 189 as the qualifier ion [43]. In addition, the retention time and characteristic ion fragments of M2 were consistent with standard amoxicilloic acid, so it was speculated that M2 was amoxicilloic acid. A weak chromatographic peak is found near the peak of M2, so M2 may have isomers. As reported, Siegrid et al. detected and characterized the 5R,6R- and 5S,6R-amoxycillic acid using Liquid Chromatography Combined with Electrospray Ionization Tandem Mass Spectrometry [36]. Liu et al. predicted the dissociated structures of *m*/*z* 323 and *m*/*z* 189 of amoxicilloic acid, both of which cleave the carboxyl group at C13, without explaining the reason for the cleavage of the carboxyl group at C13 [43]. We speculate that the carboxyl group at C21 may also be cleaved, so the fragment ions *m*/*z* 323 and *m*/*z* 189 in amoxicilloic acid may have two structures. The positions of fragment ions are shown in Figure 14 and Figure 15.

**Figure 16:**
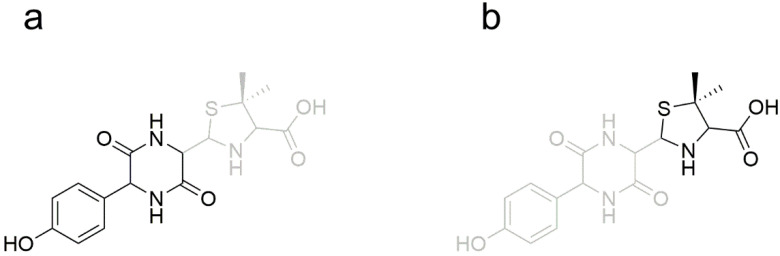
Possible structure of fragment ions in amoxicillin diketopiperazine. (**a**) Possible structure of fragment *m*/*z* 207 ion; (**b**) Possible structure of fragment *m*/*z* 160 ion.

For M3 (retention time = 6.4 min), the parent ion in the primary mass spectrometry is *m*/*z* 366 [M+H]^+^, and the molecular formula was predicted by MSC software to be C_16_H_19_N_3_O_5_S. In the secondary mass spectrometry, the main fragment ions are *m*/*z* 207, and *m*/*z* 160. It has been reported that the quantitative ion for amoxicillin diketopiperazine is *m*/*z* 160 and the qualitative ion is *m*/*z* 207 [43]. It can be seen that the retention time and the characteristic ion fragment of the metabolite are consistent with those of the amoxicillin diketopiperazine standard. Thus, the metabolite can be identified as amoxicillin diketopiperazine. Liu et al. predicted the fragment ion structure of amoxicillin diketopiperazine *m*/*z* 207, *m*/*z* 160, and the prediction result of this study is consistent with the fragment ion structure. A possible structure of the fragment ion is shown in Figure 16.

For M4 (retention time = 3.4 min), the parent ion in the primary mass spectrometry is *m*/*z* 232 [M−H]^−^, and the molecular formula was predicted to be C_8_H_11_NO_5_S by MSC software. In the secondary mass spectrometry results, the main fragments are *m*/*z* 140, *m*/*z* 64, and *m*/*z* 90. It has been reported in the literature that for sulbactam in negative ion mode, the quantitative ion is *m*/*z* 140, and the qualitative ion is *m*/*z* 64 [46,47,48]. It could be seen that the retention time and the characteristic ion fragment for the metabolite are consistent with those of the sulbactam standard. Thus, it may be determined that the metabolite is sulbactam.

## 5. Conclusions

In this study, amoxicillin, amoxicilloic acid, amoxicillin diketopiperazine, and sulbactam were identified in urine. In early literature reports, sultamicillin is excreted primarily in urine as sulbactam and ampicillin [35,38]. No heterozygous molecules or their metabolites were found in the stool samples, which is similar to the results for Sultamicillin, indicating that the heterozygous molecules are fully absorbed into the body in the intestinal tract. No intact AS hybrid molecules were found in any of the samples tested, suggesting that the hybrid molecule is broken down into amoxicillin and sulbactam in the blood and excreted in the urine by the kidneys.

Amoxicillin and sulbactam metabolites are mainly due to broken diester bonds, which is consistent with the metabolic pattern of Sultamicillin. Amoxicillin and sulbactam were detected in urine, confirming that the heterozygous molecules are broken down in vivo into amoxicillin and sulbactam.

In this study, in vivo metabolism analysis proved that AS is completely degraded in vivo, mainly producing amoxicillin and sulbactam, which play synergistic roles, significantly reducing the resistance of β-lactamase bacteria to amoxicillin.

Overall, this study provides a theoretical basis for the further development and application of amoxicillin–sulbactam hybrid molecules, and it also provides research ideas for the development of similar drugs.

## Figures and Tables

**Table 1:** Gradient elution protocol.

Time (min)	Rate of Flow (μL/min)	A (%)	B (%)
0.0 3.0	300 300	5 10	95 90
16.0	300	30	70
18.0	300	75	25
19.0	300	90	10
21.0 25.0	300 300	5 5	95 95

**Table 2:** Molecular formulas, retention times, measured molecular weights, mass errors, and fragment ions for AS and its metabolites.

Compound	Molecular Formula	Retention Time (min)	The Measured Values (Da)	The Quality of Error (ppm)	Distribution (Location)	Fragment Ions
AS	[C_25_H_31_N_4_O_10_S_2_+H]^+^	11.7	611.1478	0.32	—	594, 456, 331, 208, 114,
AMO	[C_16_H_20_N_3_O_5_S+H]^+^	2.5	366.1117	1.97	P, U, L, K	349, 321, 234, 208, 160, 114, 70
AMA	[C_16_H_22_N_3_O_6_S+H]^+^	2.2	384.1223	1.87	U	367, 323, 277, 229, 189, 160, 107
DIKETO	[C_16_H_20_N_3_O_5_S+H]^+^	6.4	366.1118	0.20	P, U, L, K	207, 160, 114
SBT	[C_8_H_10_NO_5_S−H]^−^	3.4	232.0292	0.28	P, U, L, K	232, 188, 140, 91, 64
M1	[C_16_H_20_N_3_O_5_S+H]^+^	2.5	366.1121	0.72	P, U, L, K	366, 349, 275, 208, 160, 114,
M2	[C_16_H_22_N_3_O_6_S+H]^+^	2.2	384.1228	1.49	U	384, 367, 323, 295, 229, 189, 107,
M3	[C_16_H_20_N_3_O_5_S+H]^+^	6.4	366.1125	0.19	P, U, L, K	366, 207, 160, 114,
M4	[C_8_H_10_NO_5_S−H]^−^	3.4	232.0287	0.60	P, U, L, K	232, 188, 140, 91, 64

P: plasma sample; U: urine sample; L: liver samples; K: kidney sample.

## Data Availability

The data presented in this study are available in this article.
